# Upregulation of SALL4 by EGFR activation regulates the stemness of CD44-positive lung cancer

**DOI:** 10.1038/s41389-018-0045-7

**Published:** 2018-04-25

**Authors:** Wenjing Du, Lan Ni, Baojun Liu, Ying Wei, Yubao Lv, Sujing Qiang, Jingcheng Dong, Xijun Liu

**Affiliations:** 1 Department of Integrative Medicine, Huashan Hospital, Fudan University, Shanghai, China; 2 The Institutes of Integrative Medicine, Fudan University, Shanghai, China; 3 Department of Respiratory Medicine, Zhongnan Hospital of Wuhan University, Wuhan, Hubei China; 4 Pan-Vascular Research Institute, Heart, Lung, and Blood Center, Tongji University School of Medicine, Shanghai, China

## Abstract

The transcriptional factor SALL4, an important stem cell regulator, is expressed in hematopoietic stem cells and various malignancies, but its role in EGFR-mutated NSCLCs has not been studied yet. Here, we report that the expression of Sal-like protein 4 (SALL4), was significantly higher in EGFR mutated lung tumors than in non-tumor tissue. SALL4-high lung cancer patients had poorer prognosis after surgery than SALL4-low patients. The expression of SALL4 could be induced by the activation of EGFR through the extracellular signal-regulated kinase 1/2 (ERK1/2) signaling pathway. The knockdown of SALL4 expression could suppress spheroid formation and the expression of lung cancer stem cell marker CD44. More interestingly, the knockdown of SALL4 expression could suppress the migration, invasion, and metastasis of the lung cancer cells and significantly increase the sensitivity of EGFR mutated cells to Erlotinib. These results suggest that SALL4 may be a novel potential therapeutic target for the diagnosis and treatment of lung cancer.

## Introduction

Lung cancer is marked by genetic and histopathological heterogeneity, and remains the leading cause of cancer deaths globally^1^. The phenotypic diversity of cancer cells has traditionally been explained by changes in genetic and nongenetic factors. The mechanisms underlying tumorigenesis, medicinal resistance, and recurrence are not clearly established. Cancer stem cells may provide a powerful explanation for tumor heterogeneity^2^. There is increasing evidence that cancer is maintained by a subset of tumor cells with stem/progenitor cell characteristics^3^. Recent studies also showed that this small subset of tumor cells can generate tumor more efficiently in vivo and less vulnerable to chemo/radiation-resistant than other tumor cells^4^.

The SALL4 encoded a zinc finger transcription factor, is a part of the Spalt-like (SALL) gene family and the DNA sequence is homology to Drosophila gene spalt (sal)^5^. SALL4 is involved in proliferation of pluripotent stem cells and maintenance of pluripotent state through interactions with OCT4, SOX2, and KLF4^6,7^. Recently, Xiong et al. reported that SALL4 involve in the regulation of stemness state and survival in normal stem cells^8–10^. Moreover, SALL4 expression is also reported in numerous malignancies, such as endometrial, breast, lung, and liver carcinomas, and leukemia. SALL4 transgenic mice could spontaneously develop acute myeloid leukemia (AML)^5,8,11–15^.

Non-small-cell lung cancer (NSCLC) accounts for over 80% of all human lung cancer that is the leading cause of cancer death around the world^18,19^. The epidermal growth factor receptor (EGFR) gene is the most common oncogenic driver mutant genes^20^. Inhibitors of EGFR signaling, such as Erlotinib and Gefinitib could significantly repress tumor growth, but the drug resistance is still a problem during treatment. Previous studies have shown that SALL4 was aberrantly expressed in lung cancer tissues and not in normal controls^21,22^. In this study, we not only demonstrated that SALL4 was highly in lung cancer tissues, but also found that aberrant expression of SALL4 was associated EGFR mutation in NSCLC was essential for the cell stemness of EGFR mutant-driven NSCLC cells.

## Results

### SALL4 expression is significantly correlated with poor survival

**Fig. 1:**
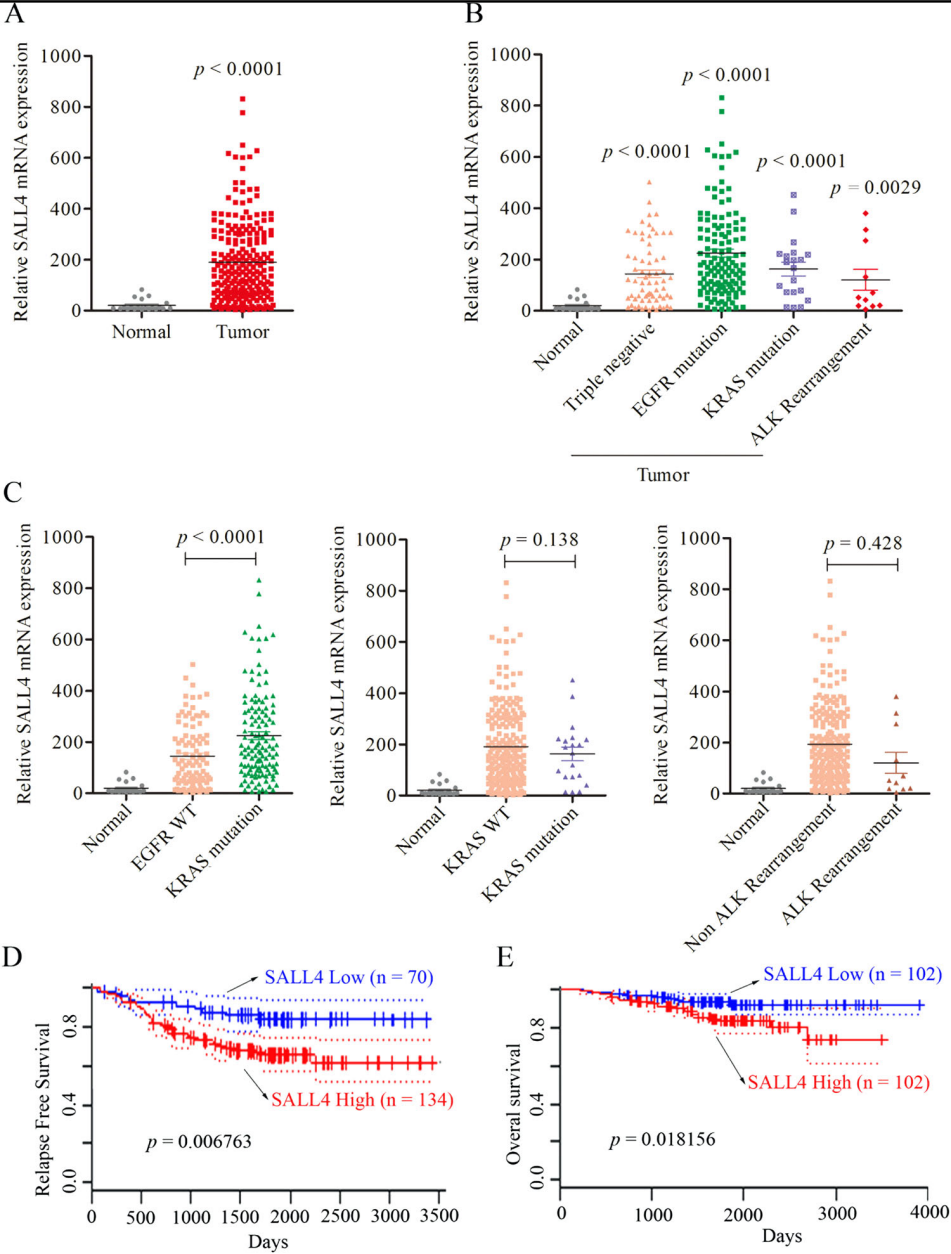
Association of high SALL4 expression with activated EGFR mutations in lung cancer tissues. **a** Microarray analysis of SALL4 expression in normal (*n* = 20) and lung tumor (*n* = 226). **b** The highest expression of SALL4 in EGFR mutation in comparison with KRAS mutation, ALK-rearrangement, and normal WT tissues. The average value of SALL4 expression is shown in the graph. **c** SALL4 upregulation is specifically induced by EGFR mutation, but not EGFR WT, KRAS mutation and ALK rearrangement in human lung cancer. **d, e** Kaplan–Meier survival analysis with Log-rank from the PrognoScan database. Recurrence-free survival and overall survival of SALL4-low and SALL4-high NSCLCs was analyzed. The *P*-value for the difference between the two curves were determined by the log-rank test

A panel of tissue microarrays (*n* = 226) was used to detect the stem cell marker SALL4 expression in normal and NSCLC tissues. Normal lung tissues (*n* = 20) served as controls. SALL4 expression showed significant differences between normal and tumor tissues of NSCLCs (Fig. 1a). To analyze the correlation between the expression of SALL4 and EGFR mutation, microarray analysis of lung cancer cases was performed and results showed that the SALL4 expression was highest in lung cancer with EGFR mutations among tumor and non-tumor cases (Fig. 1b). Three types of oncogene activation were used to investigate their correlation with SALL4 expression. More interestingly, the SALL4 expression in EGFR-mutant lung cancer was significantly higher than that in lung cancer tissue with wild-type EGFR (Fig. 1c). This results showed that SALL4 upregulation is resulted in EGFR activation, but not KRAS mutation and ALK rearrangement. Finally, our results strongly indicated the EGFR activation could specifically induce the expression of SALL4 in lung cancer and also suggest a positive association between SALL4 expression and EGFR mutation. Then, we also evaluated the survival outcome of all lung cancer cases by Kaplan–Meier survival analysis indicated that SALL4-high NSCLCs had lower overall and recurrence-free ten-year survival ratio in comparison to that of SALL4-low NSCLCs from the PrognoScan database (Fig. 1d, e). These data suggest a positive correlation between the expression of SALL4 and EGFR mutation.

### EGF stimulates SALL4 expression and SALL4 is down-regulated by EGFR/ERK inhibition

**Fig. 2:**
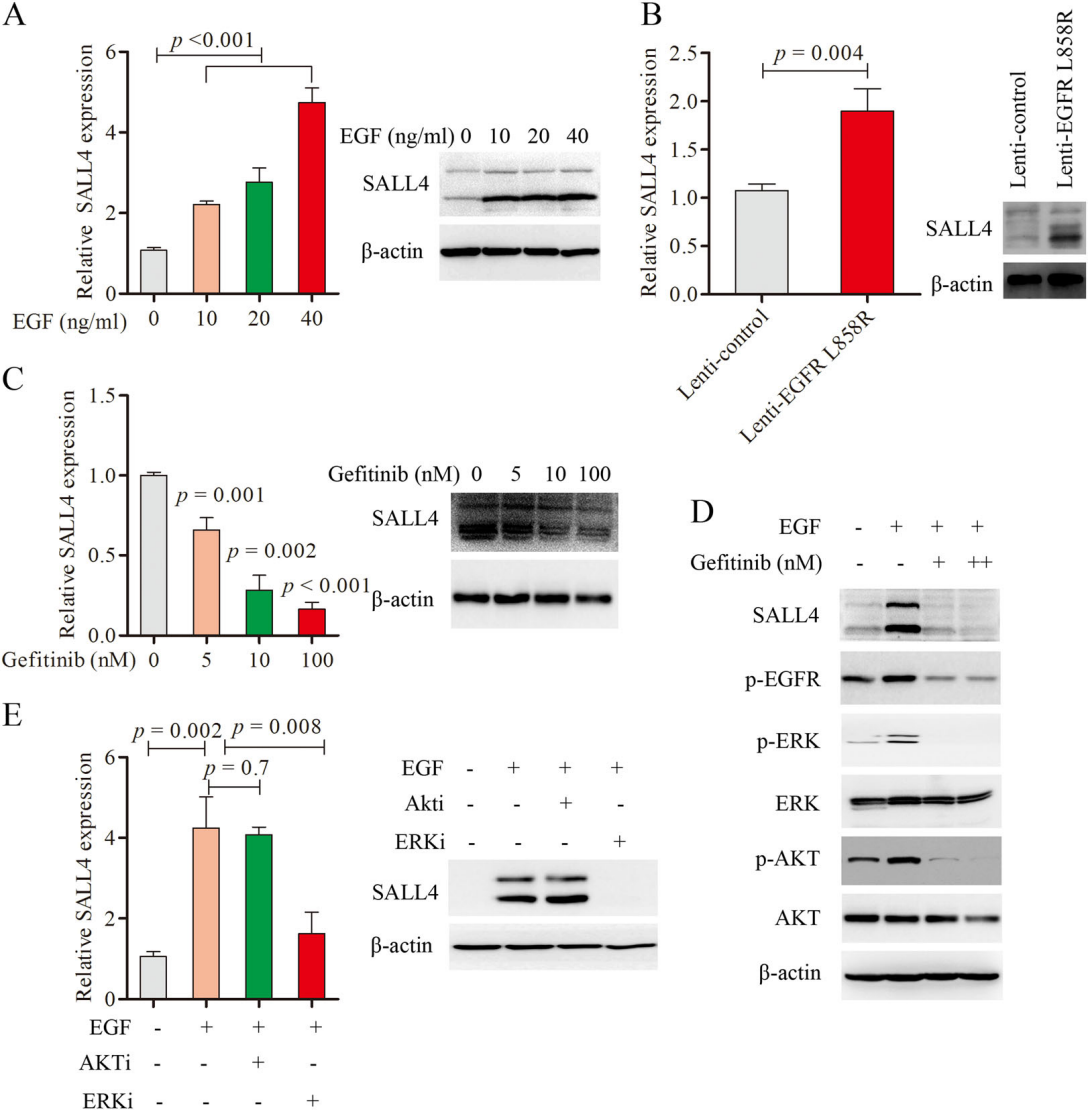
EGFR signaling activated by the ligand EGF stimulation and EGFR L858R point mutation induced the expression of SALL4 through ERK1/2 pathway. **a** qRT-PCR analysis of SALL4 expression in Beas-2B cells treated with different dose of EGF (0, 10, 20, and 40 ng/ml) for 30 min and the protein level of SALL4 was detected by Western blotting. **b** qRT-PCR and western blotting analysis of SALL4 expression in Bease-2B cells infected with Lenti-control and -EGFR L858R. **c** qRT-PCR and Western blot analysis of SALL4 expression in PC9 treated with different doses of Gefitinib (0, 5, 10, 100 nM) for 48 h respectively. **d** The protein level of SALL4, p-EGFR, p-ERK, ERK, p-AKT, and AKT in PC9 cells treated with Gefitinib (100, 500 nM) for 48 h, and β-actin was selected as a control. **e** The mRNA and protein level of SALL4 in PC9 cells, which were treated with 1 μM ERK inhibitor (SCH772984) and 2 μM AKT inhibitor (MK-22062HCL) for 72 h. Representative results from three independent experiments are shown (mean ± s.d.)

To illustrate the mechanism of EGFR activation activates the expression of SALL4, we used EGF as a ligand of EGFR to activate wild type EGFR in Beas-2B cells. After 24 h, the treatment of EGF led to a dramatical increase of SALL4 mRNA and protein level in a dose-dependent manner (Fig. 2a). Then whether constitutive EGFR activation regulates the expression of SALL4. The plasmid harboring EGFR with L858R point mutation was used to produce high EGFR activity, and then this results indicated that the mRNA and protein levels of SALL4 elevated dramatically in Beas-2B-Lenti-EGFR L858R cells in comparison with Beas-2B-Lenti-control (Fig. 2b). These results indicated that SALL4 expression was regulated by activated EGFR. We also observed the effect of Gefitinib, an EGFR inhibitor on SALL4 expression. The result showed that Gefitinib significantly inhibit the expression of SALL4 in PC9 cells with EGFR-19del in a dose-dependent manner (Fig. 2c). To determine which signaling pathway was involved in EGFR activation-induced SALL4 expression in PC9 cells, we detected the classic downstream pathways of EGF/EGFR. The results showed that the ERK1/2 and AKT pathways were activated by EGF and suppressed by EGFR-TKI (Fig. 2d). Our data indicate that ERK1/2 and AKT signaling may be involved in regulation of SALL4 by EGFR activation. To further explore the mechanism, we use inhibitors of ERK1/2 and AKT to block the downstream signaling pathways of EGFR in PC9 cells. The results showed that the significant attenuation of SALL4 expression was induced by EGFR activation after treatment with ERK1/2 inhibitor, but not AKT inhibitor (Fig. 2e). Collectively, these results indicate that SALL4 expression was induced by EGFR activation and that the ERK1/2 pathway plays a key role in the up-regulation of SALL4 in EGFR mutated NSCLCs.

### SALL4 expression is primarily expressed in CD44-positive NSCLCs

**Fig. 3:**
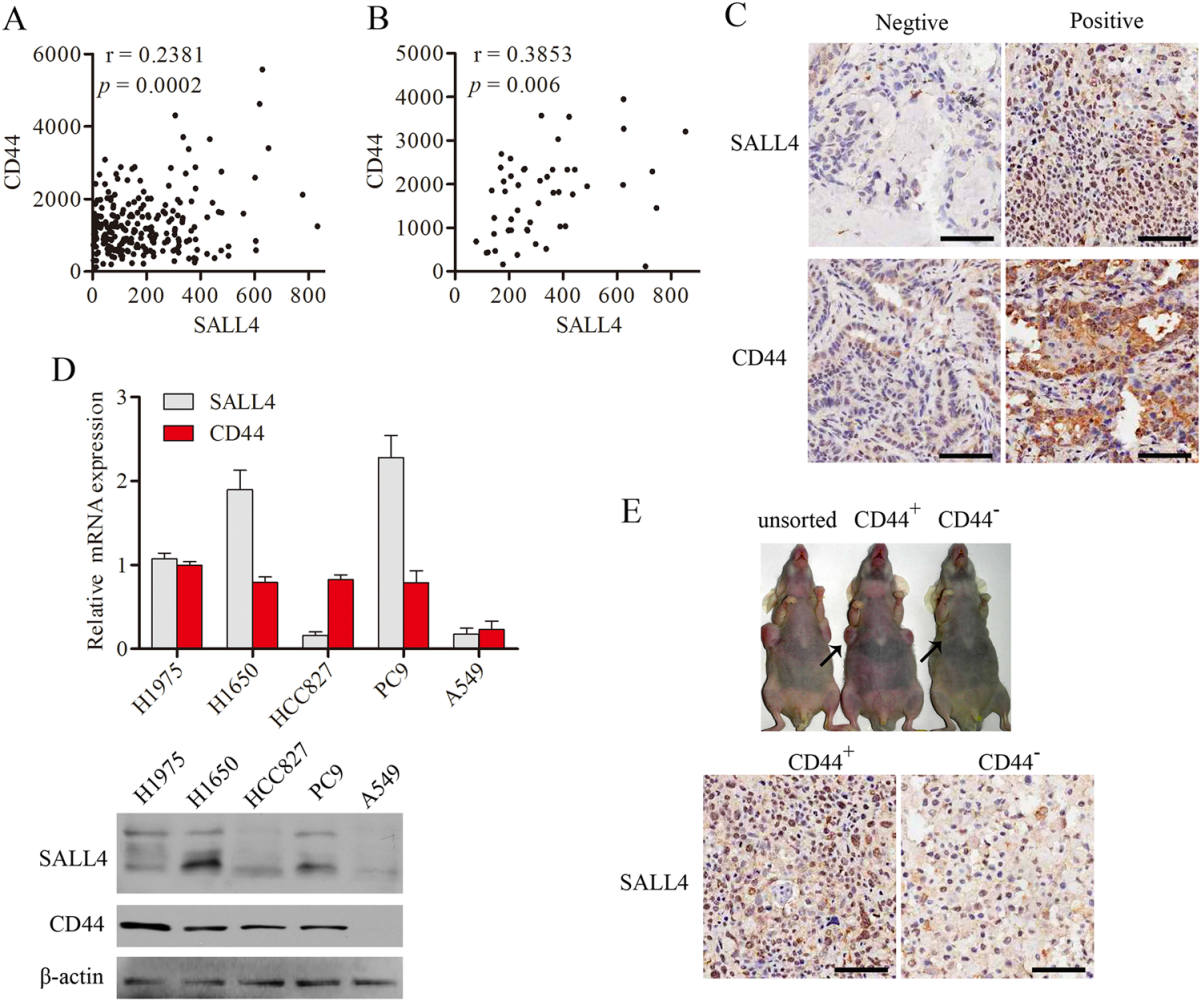
Coexpression of the transcription factor SALL4 and a cancer stem cell marker CD44 in human lung cancer. **a** Microarray analysis proved that gene expression levels of CD44 were positively correlated with those of SALL4, as shown by Spearman’s correlation coefficient. **b** qRT-PCR analysis of SALL4 and CD44 expression in human lung cancer tissues (*n* = 49), as shown by Pearson’s correlation coefficient. **c** The representative images of SALL4 or CD44 immunostaining (Bar = 100 μm). **d** The gene expression levels of SALL4 were determined by qRT-PCR (upper panel) and Western blotting (lower panel) in different NSCLC cell lines. **e** The tumorigenicity assay of CD44 positive (+) and negative (−) −PC9 cells achieved by 2 × 10^5^ cells was subcutaneously injected in nude mice (upper panel). IHC analysis of xenografts from recipient mice showed SALL4 expression in cell nuclear distribution (lower panel) (Bar = 100 μm) (mean ± s.d.)

Previous data showed that the transcriptional factor SALL4, as an important stem cell regulator, was known to be expressed in hematopoietic stem cells and other solid tumors, but its role in EGFR mutated NSCLCs have not been studied at present. Recent studies suggested that CD44 appeared to be a potential lung cancer stem cell marker. Thus, we focused on the expression of SALL4 in CD44^+^-NSCLC. Firstly, it is found that SALL4 expression was positively correlated with CD44 expression in NSCLC as evaluated by microarray analysis (Fig. 3a). To confirm this finding, we further examined SALL4 mRNA expression in NSCLC tissues by RT-PCR, and also was showed the positive correlation between SALL4 and CD44 expression (Fig. 3b). In the meantime we performed IHC analysis of SALL4 and CD44 in NSCLC tissues. We detected a stronger nuclear expression of SALL4 in CD44^+^-NSCLC than CD44^−^-NSCLC (Fig. 3c). Because SALL4 expression was strong positively correlated with CD44 expression in primary NSCLC tissues, we also measured the expression of SALL4 and CD44 in NSCLC cell lines by qRT-PCR and western blotting analysis (Fig. 3d). We further evaluated the correlation between the expression of SALL4 and CD44 using the marker-selected NSCLC cells to initiate in vivo tumor by subcutaneous injection into nude mice. As showed in Fig. 3e, CD44^+^ and unsorted PC9 cells were able to initiate bigger tumor size than CD44^−^-PC9 cells after 45 days. Then, we performed IHC analysis of tumors surgically resected from PC9 xenografts. The IHC data showed that the density of nuclear staining of SALL4 in the tissue from CD44^+^-PC9 cells was stronger in comparison with the density from CD44^−^-PC9 cells. These findings indicate that SALL4 is activated in CD44^+^-NSCLC cancer stem cells.

### SALL4 regulates stemness of NSCLC cells

**Fig. 4:**
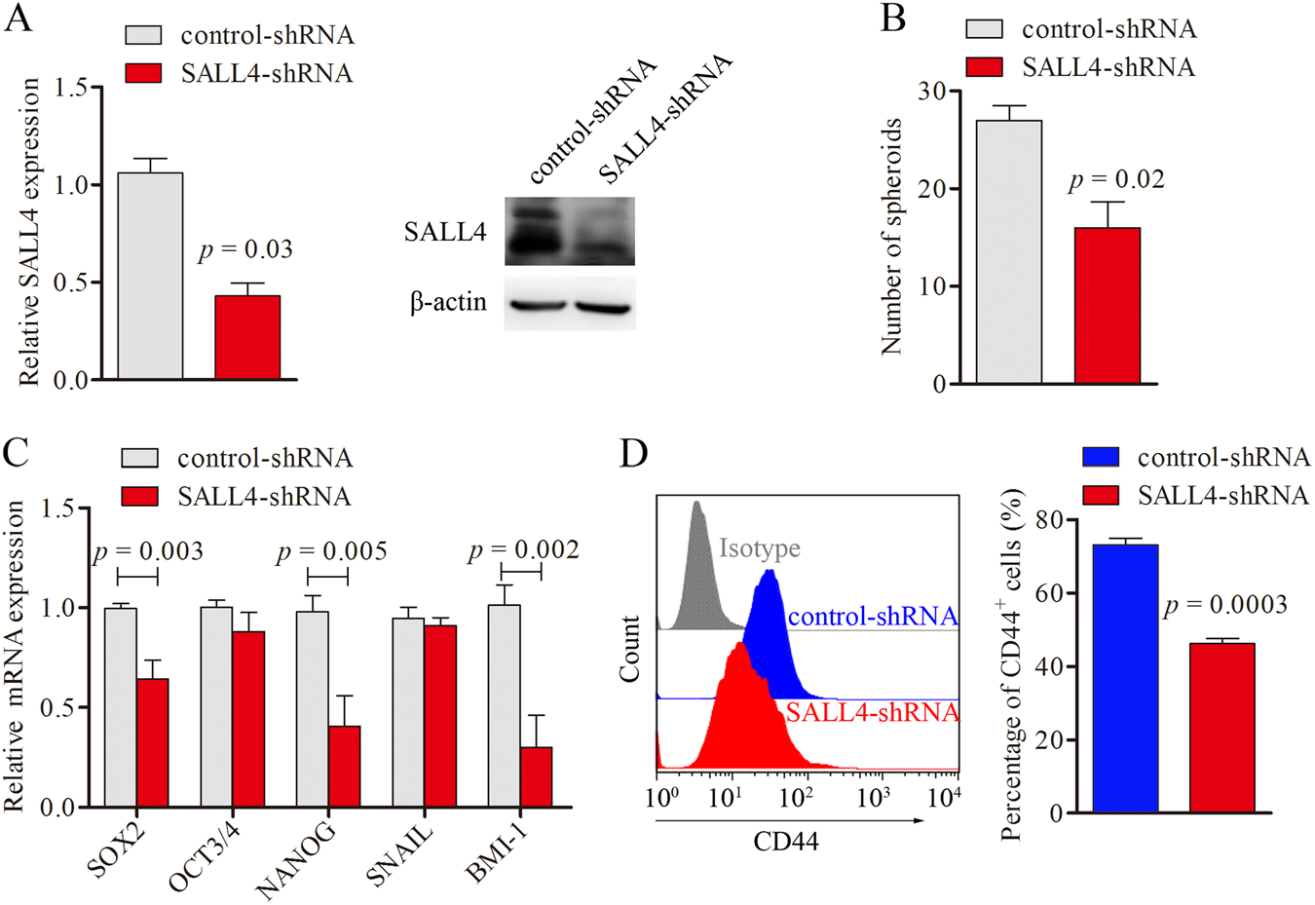
SALL4 knockdown reduced the amount of spheroid formation in vitro and spheroid formation and the population of CD44^+^-PC9 cells. **a** Knockdown of SALL4 by shRNA was detected by qRT-PCR and western blotting. **b** Spheroid formation assay of PC9 cells were infected with Lenti-control or SALL4 shRNA. **c** qRT-PCR analysis of SOX2, OCT3/4, NANOG, SNAIL, and BMI-1 expression in PC9 cells infected with Lenti-control or shRNA. **d** The expression of CD44 were determined by flow cytometry in PC9 cells infected with Lenti-control or SALL4 shRNA. Representative data from three independent experiments are shown (mean ± s.d.)

To investigate the role of SALL4 in NSCLC cancer stem cells, the SALL4 knockdown was evaluated in PC9 cells with the highest SALL4 expression. A loss-of-function study was performed using the Lenti-shRNA virus tool. SALL4 mRNA expression was lower in PC9 cells transfected with Lenti-SALL4 shRNA than in controls, as determined by qRT-PCR. The SALL4 protein level was reduced significantly, as determined by Western blot analysis (Fig. 4a). Knockdown of SALL4 resulted in the reduction of spheroid formation capacity with decreased expression of many pluripotency genes in PC9 cells, including SOX2, OCT3/4, NANOG, SNAIL, and BMI-1 (Fig. 4b and c). Importantly, the surface marker CD44 of stem cells in PC9 cells transfected with Lenti-SALL4 shRNA was much lower than in the Lenti-control shRNA by FACS assay (Fig. 4d). All in all, these results suggest that SALL4 is involved in inhibiting the spheroid formation capacity and reducing the expression of lung cancer stem cell markers and pluripotency transcriptional factors.

### SALL4 knockdown reduces lung cancer migration, invasion, and metastasis

**Fig. 5:**
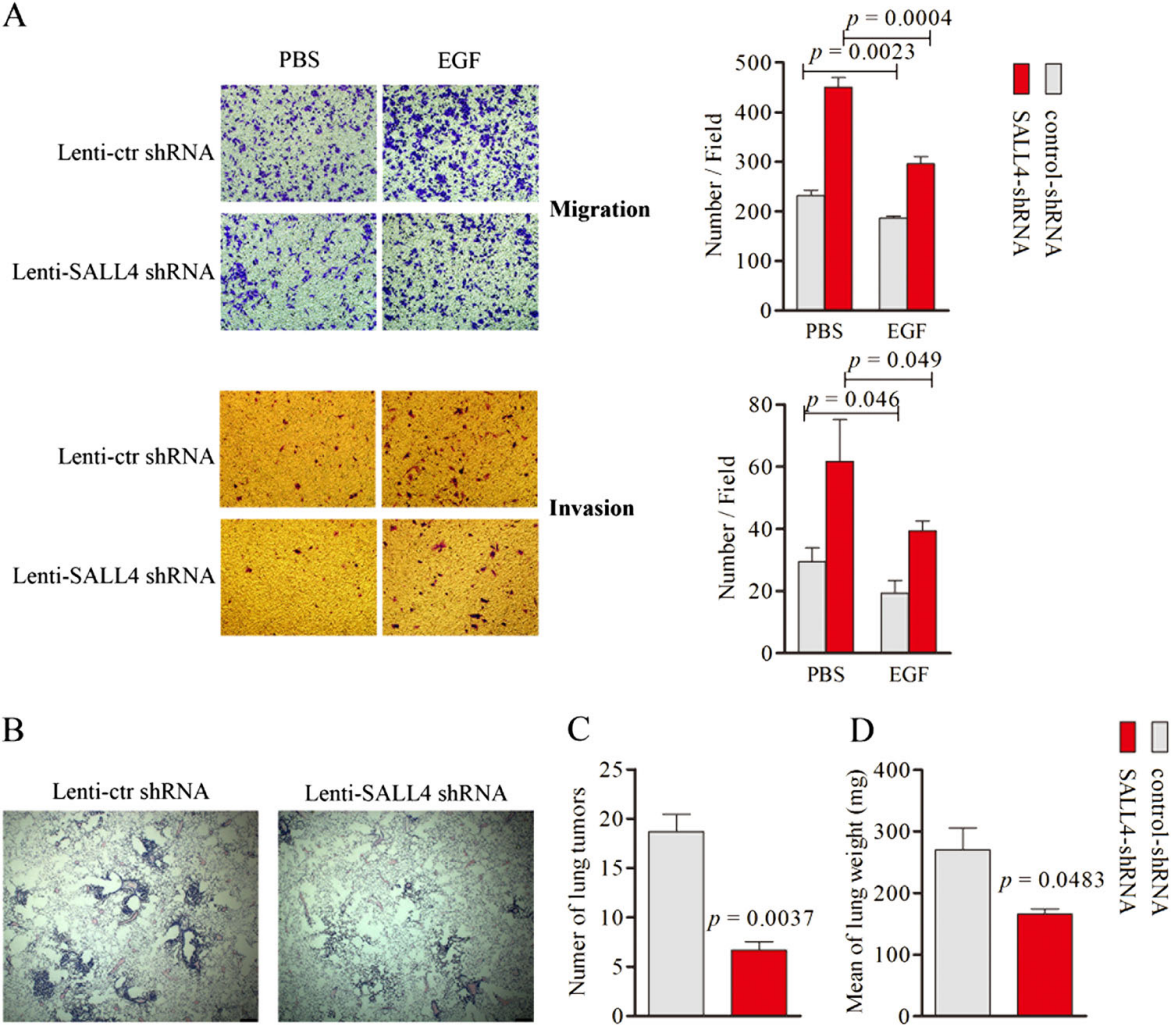
Silencing of SALL4 decreases lung cancer cells migration, invasion in vitro and metastasis in vivo. **a** Representative cell migration and invasion capacities were measured after the Bease-2B cells were stained with crystal violet (mean ± s.d.). **b** Lung tissues from mice injected with Lenti-control or SALL4 shRNA-treated PC9 cells. Representative metastasis capacities were evaluated after the lung tissues and stained with hematoxylin and eosin. Bar = 200 μm. **c** Number of left lung metastases in which the long axis in Lenti-control or SALL4 shRNA-treated PC9 groups was determined at 45 days after intravenous injection. **d** The weight of left lungs was determined in each group. Data are means ± s.d. for 5 mice per group

To study the effects of SALL4 on cell migration, invasion, and metastasis. We treated Beas-2B cells, a human bronchial epithelium immortalized cell line, with EGF and Lenti-SALL4 shRNA. Expression of SALL4 was inhibited in Beas-2B cells infected with Lenti-SALL4 shRNA compared with the control evaluated by western blotting (Supplementary Fig. 1). The results showed that cell migration and invasion could be stimulated by EGF and significantly suppressed by SALL4 knockdown (Fig. 5a, b), suggesting that SALL4 plays an important role in EGF-stimulated cell migration and invasion. To observe the effects of SALL4 on metastatic activities, we transfected the PC-9 cells with Lenti-SALL4 shRNA and transplanted them into nude mice. The results of histological analysis showed that the metastatic activities of Lenti-shRNA transfected cells were significantly reduced than that of control cells (Fig. 5c, d). These data indicates that SALL4 regulated EGF-mediated cell motility, invasion, and metastasis.

### SALL4 expression affects the sensitivity of EGFR mutation-positive cells to Erlotinib

**Fig. 6:**
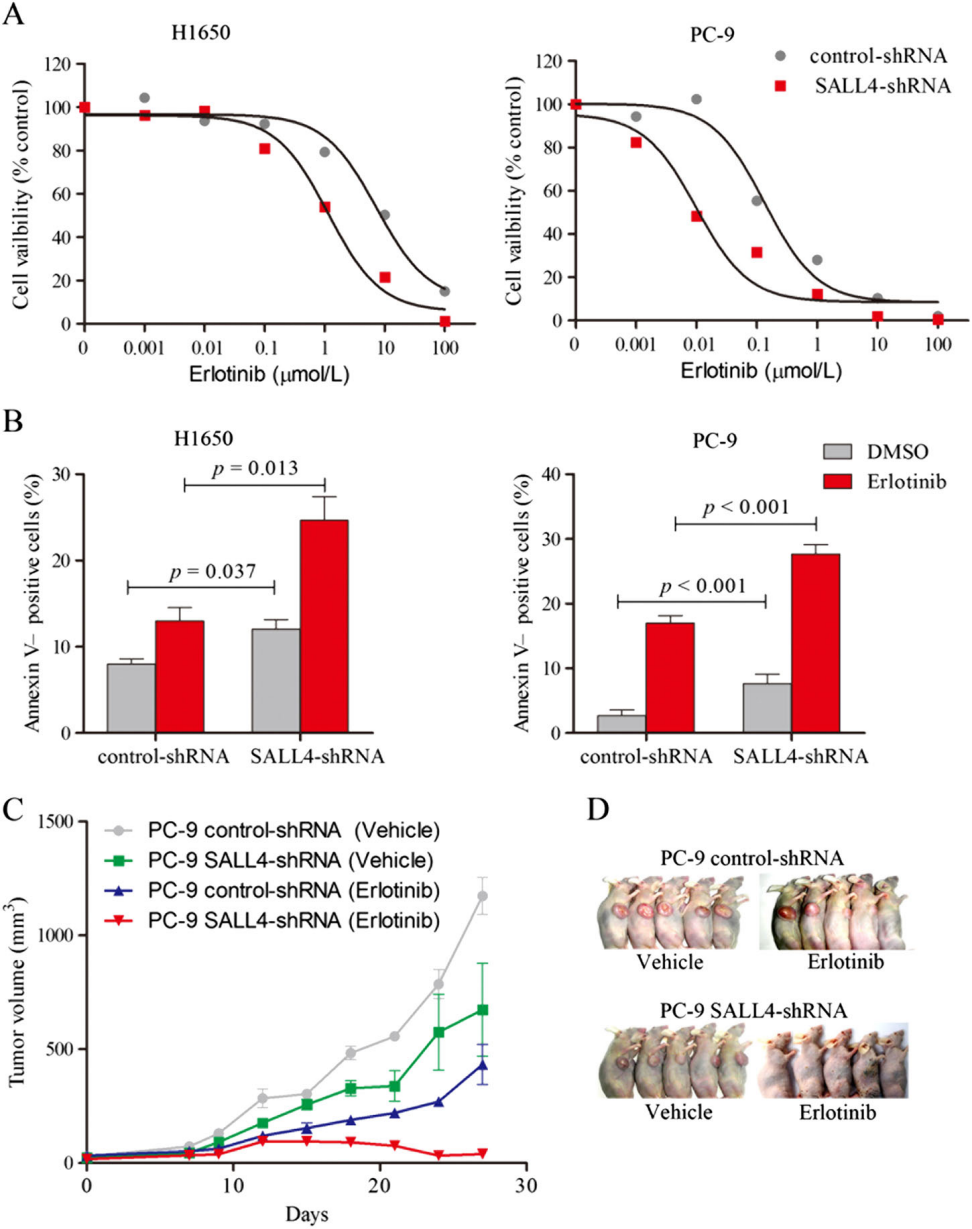
SALL4 knockdown results into alterations in drug sensitivity of NSCLCs response to EGFR-TKIs. **a** PC9 cells infected with Lenti-control or SALL4 shRNA were cultivated in medium with and without Erlotinib for 72 h, then the cell viability was assessed. **b** Cells were kept in medium with and without Erlotinib (100 nM) for 48 h, and the apoptotic cells was measured by staining with Annexin V-PI by FACS. Data are representative of three independent experiments. **c** Nude mice with xenografts established by subcutaneous injection of PC9 cells infected with Lenti-control or SALL4 shRNA were treated with vehicle or Erlotinib (10 mg/kg). Data are means ± s.d. for 5 mice per group. **d** Representative imaging of mice injected PC-9 cells treated with or without Erlotinib

To investigate the effects of SALL4 on Erlotinib resistance, knocked down the SALL4 expression in H1650 and PC-9 cells. Results showed that knockdown of SALL4 increase the sensitivity of H1650 and PC-9 cells to the Erlotinib (Fig. 6a) and promote the Erlotinib-induced apoptosis (Fig. 6b). Similar results were also observed in EGFR-mutant cells in vivo (Fig. 6c, d). Collectively, these results suggested that knockdown of SALL4 enhances the sensitivity of EGFR mutation-positive tumor cells to Erlotinib in vivo.

## Discussion

The EGFR gene is one of the most oncogenic driver mutations. EGFR molecular aberrations were found to be potential predictive biomarkers for response to target therapy with tyrosine kinase inhibitors in NSCLs^25,26^. Molecularly targeted therapies have expanded a new era of personalized medicine for the treatment of lung cancer. The development of effective therapeutic strategies that target the EGF/EGFR signaling pathway is urgently required. Currently, two EGFR-(TKIs), Gefitinib and Erlotinib, are the front-line therapies in NSCLC patients with mutant versions of EGFR. However, NSCLCs can easily produce drug resistance on EGFR-TKIs and recurrence^27^. More precise and effective treatment of patients with EGFR mutant NSCLC requires an in-depth understanding of the mechanisms of primary and recurring tumor-acquired resistance to EGFR-TKIs.

Previous studies have demonstrated that SALL4 is aberrantly expressed in a variety of human cancer types and significantly closely correlated with the prognosis of patients. SALL4 is also required for cell proliferation, invasion, and maintenance of pluripotency of stem cells in embryonic stem cell and malignantly transformed stem cells, such as leukemia, breast cancer, and liver cancer. Jia et al. found that SALL4 expression was highest in lung cancer with EGFR mutation, but not KRAS or EML4-ALK mutation. However, this conclusion was necessary but not sufficient summary representation of the association between SALL4 expression and EGFR mutation^17^. In this study, we found that there was highest SALL4 expression in NSCLCs harboring EGFR mutations than in tissues with other oncogenic mutations, such as KRAS and ALK. This results showed that SALL4 upregulation is induced by EGFR mutation, but not EGFR WT, KRAS mutation and ALK rearrangement in human lung cancer. Consistent with published results by other groups, we also found that SALL4 is overexpressed in NSCLC patients with EGFR mutation. Moreover, the association between high SALL4 expression and poor patient survival is also proved in lung cancer, gastric cancer, endometrial cancer, and myelodysplastic syndrome^33,34^. In our study, it is found that EGF induce SALL4 expression in wild-type EGFR Beas-2B cells. The induction of SALL4 by constituting EGFR activation was confirmed in Beas-2B with EGFR L858R point mutation. Conversely, the EGFR-TKI Gefitinib reduced the expression of SALL4 in PC9 cells with activated EGFR mutant. To explore the mechanism of SALL4 induction by activated EGFR, the downstream pathway ERK1/2, which is involved in the cell migration, invasion, survival, and death, was evaluated. In the present study, the induction of SALL4 expression by EGF/EGFR signaling mediated by ERK1/2 signaling pathway. However, blocking AKT did not alter the expression of SALL4. Recently, Tricker EM et al. found that concomitant inhibition of ERK1/2 by MEK inhibitor Trametinib prevented ERK 1/2 reactivation, resulted in EGFR inhibitors-induced antitumor effects and inhibited the emergence of resistance in PC-9 cells^28^. Previous studies has suggested that SALL4 increase the drug transporters ABCG2 and ABCA3 expression, but it has not been sufficiently analyzed in clinical tissues^16^. In addition, Nozomi Yanagihara et al. report that the expression of SALL4 is a resistance factor against conventional chemotherapeutic drugs such as CDDP, CBDCA, and PTX. However, it’s unclear whether SALL4 inhibition can alter the sensitivity to all types of anticancer drugs or might be a common resistance factor in lung cancer^31^. Our results indicate that SALL4 expression is associated with EGFR mutation and SALL4 inhibition can increase the sensitivity of EGFR TKIs in lung cancer.

CD44 has been defined as a potent cancer stem marker in diagnosis of NSCLC, because the xenograft initiation capacity of CD44^+^-NSCLC cells is much greater than in CD44^−^-NSCLC cells^24,29^. Our data indicated a positive correlation between SALL4 and CD44 expression in human NSCLCs. CD44^+^-PC9 cells have a much higher expression of SALL4 than CD44^−^-PC9 cells. The capacity of the subset of CD44^+^-PC9 cells was inhibited by knockdown of SALL4 expression. Meanwhile, the knockdown of SALL4 affected the tumorgenesis of PC9 cells. Collectively, these data suggest that SALL4 plays an essential role in maintenance of stemness in CD44^+^-NSCLCs, and blocking SALL4 might be helpful for targeted treatment of CD44^+^-NSCLCs. We will investigate the downstream target genes of SALL4, to find out which genes are directly or indirectly taken part in the SALL4 regulation of NSCLC cancer stem cells.

Rational targeting of the EGFR family activity to control these signaling pathways in cancers has been successful in improving outcomes of many types of cancer. However, patients initially responding to targeted therapy always develop resistance to the drugs. In recent years, many studies indicate cancer stem cells (CSCs), also known as cancer-stem-like cells that is a minor subpopulation of cells exhibited stem cells properties of self-renewal and differentiation^30,32^. Our data showed that inhibition of EGFR pathway resulted in the downregulation of SALL4, and knockdown of SALL4 affected the self-renewal properties of CSCs. The enrichment of cells with CSCs markers (CD44^high^/CD24^low^) and phenotypes in Erlotinib resistant NSCLC cell lines has been reported^32,35^. These observations suggest that CSCs was also selected during prolonged exposure to EGFR TKIs and contribute significantly to drug resistance and tumor relapse. Recent studies demonstrate that mutations of EGFR, c-MET, and K-RAS was associated with drug resistance^36–39^. The better understanding of the characteristics of cancer stem cells will facilitate development of novel therapies to overcome cancer drug resistance.

## Materials and methods

### Clinical NSCLC specimens

A total of 49 lung cancer tissues were obtained from patients from 2012 to 2014 at Zhongnan Hospital of Wuhan University, China. The samples were collected from surgically resected specimens, rapidly snap-frozen in liquid nitrogen. All experiments were approved by the authors’ institutional Ethics Committee of Wuhan University’s Zhongnan Hospital and Huashan Hospital Affiliated to Fudan University.

### Cell lines and reagents

The NSCLC cell lines HCC827, H1650, H1975, PC9, A549, and normal lung tissue cell Beas-2B were cultured in DMEM (Life Technology) supplemented with 10% FBS (Gibco), incubated at 37 °C with 5% CO_2_ in humidified incubator. EGF, FGF, Erlotinib, and Gefinitib were purchased from R&D Systems and Sigma-Aldrich respectively. ERK inhibitor (SCH772984) and AKT inhibitor (MK-22062HCL) were obtained from Selleck Chem. Expression plasmids for point mutation EGFR L858R were obtained from Addgene (Plasmid #11012). Lentivirus-mediated delivery of shRNA directed against two isoforms of SALL4 was performed as previously described^11^.

### Microarray data analysis

Gene expression data of lung adenocarcinomas were collected from the GEO database (accession number: GSE31210)^23^. The specific relationship between SALL4 expression and overall survival and relapse-free survival was analyzed using the minimum *P*–value approach in PrognoScan. Comparison of two survival curves were analyzed by the log-rank test.

### Western blotting and immunohistochemistry (IHC)

Protein extraction from cells were isolated in RIPA lysis buffer (25 mM Tris-HCl (pH 7.4), 10% (v/v) glycerol, 150 mM NaCl, 2 mM EDTA, 1% (v/v) phosphatase inhibitor cocktail II (Thermo Scientific), and 1% (v/v) protease inhibitor cocktail III (Thermo Scientific)). Then protein concentrations was determined using a Thermo Scientific Pierce BCA Protein Assay Kit and denatured in 1× Laemmli’s gel loading buffer. Cellular protein was separated by SDS-PAGE and transferred to PVDF membranes, then probed with primary antibodies against SALL4, p-EGFR (Tyr1068), ERK1/2 (L34F12), p-ERK1/2 (Thr202/Tyr204), AKT (pan) (C67E7), p-AKT (Ser473), and β-actin (Cell Signaling). Signals were detected using a chemiluminescence kit (Thermo Scientific). The sections of human lung cancers were subjected to hematoxylin-eosin staining and immunohistochemical staining using a VECTASTAIN ABC Kit (rabbit/mouse IgG) with anti-SALL4 and anti-CD44 (Cell Signaling) antibodies.

### Flow cytometry analysis and fluorescence-activated cell sorting

PC9 cells were labeled with anti-CD44-fluorescein isothioyanate (FITC) specific mouse antibody and isotype-matched mouse immunoglobins served as negative controls (BD Bioscience). Dead cells and debris were first gated out using Propidium iodide (PI) based on cell size and internal complexity. At least 10,000 cells were selected for each analysis. Under sterilized conditions, cells labeled by anti-CD44-fluorescein isothioyanate (FITC) were sorted by BD FACS cell sorter. The cell viability was determined using Annexin V/PI Apoptosis Detection kit (BD Biosciences, U.S.). All data were analyzed using Flowjo software^24^.

### Spheroid culture and in vitro transplantation

To evaluate sphere-formation potential, PC9 cells were sorted (CD44-positive and CD44-negative) and seeded in DMEM medium supplemented with human EGF (20 ng/ml) and bFGF (10 ng/ml) in 6-well plates at a density of 2 × 10^3^ cells. The number of spheroids was counted at 14 days after seeding. To assess the ability of in vivo tumor-initiating properties of CD44^+^ cells, CD44^−^ cells or unsorted PC9 cells (2 × 10^5^/100 μl) were transplanted into nude mice. The tumor volume was measured weekly. When the biggest tumor volume reached about 300 mm^3^, all the mice were sacrificed and tumor tissues were harvested for the following experiments. Cells (2 × 10^6^) were s.c. injected into 6-week-old nude mice. One week after injection, mice were randomly assigned to four groups (*n* = 5 mice/group) that treated with either vehicle, or 10 mg/kg Erlotinib by oral gavage (5 days a week). Tumor volume (width^2^ × length/2) was determined every three days. The procedures of animal experiment were performed according to the guide for the Care and Use of Laboratory Animal and with Approval of the Animal Care and Use Committee of Wuhan University.

### Quantitative real-time PCR

Total RNA was isolated using TRIZOL reagent (Life Technology), and then reverse-transcribed into cDNA by using M-MLV reverse transcriptase (Life Technology) with oligo random hexzmers. Finally, the cDNA was subjected to real-time PCR analysis in an ABI7500 sequence detection system. The primers used were as follows: SALL4-FW, 5′-TACCACCAAAGGCAACTTAAAG-3′; SALL4-RW, 5′-GTTCTCGATGGCCAACTTCC-3′. Relative quantitation of mRNA was determined using the ΔC_t_ method with GAPDH as the endogenous control.

### Migration, invasion, and growth inhibition assays

The filter of transwell system with 8 μm pores size (Corning Incorporated) was used for migration assays and Matrigel-coated filters were used for invasion assays. The shRNA-transfected cells selected with puromycin were seeded into inserts, and then the cells that migrated to the underside of the membrane were fixed and stained with crystal violet solution. Cells on the top were erased by cotton swab, and the images were acquired using an Olympus IX71 microscope. Cells were seeded to 96-well plates and cultured for 12 h before 72 h of exposure to various concentration of Erlotinib, and the cell viability was measured by MTS assay.

### Statistical analysis

Quantitative data were expressed as the mean ± s.d. using Student’s *t* tests, and Spearman’s correlation coefficient and Kaplan–Meier survival analysis were performed with GraphPad Prism for comparison of test groups assayed by qRT-PCR and FACS analysis. The significance of differences was evaluated with the Student *t* test (unpaired 2-tailed). *P* < 0.05 was defined as statistically significance.

## Electronic supplementary material

Supplementary Figure legend

Supplementary Figure S1

## References

[1] Ardalan Khales S (2015). SALL4 as a new biomarker for early colorectal cancers. J. Cancer Res. Clin. Oncol..

[2] Basak SK (2013). The CD44(high) tumorigenic subsets in lung cancer biospecimens are enriched for low miR-34a expression. PLoS One.

[3] Brugger W, Thomas M (2012). EGFR-TKI resistant non-small cell lung cancer (NSCLC): new developments and implications for future treatment. Lung. Cancer.

[4] Chou T, Finn RS, Garon EB (2012). Expanding options for EGFR targeting in lung cancer. Transl. Lung Cancer Res..

[5] Dahabreh IJ (2010). Somatic EGFR mutation and gene copy gain as predictive biomarkers for response to tyrosine kinase inhibitors in non-small cell lung cancer. Clin. Cancer Res..

[6] Engelman JA (2007). MET amplification leads to gefitinib resistance in lung cancer by activating ERBB3 signaling. Science.

[7] Eramo A, Haas TL, De Maria R (2010). Lung cancer stem cells: tools and targets to fight lung cancer. Oncogene.

[8] Fujimoto M (2014). SALL4 immunohistochemistry in non-small-cell lung carcinomas. Histopathology.

[9] Fulawka L, Donizy P, Halon A (2014). Cancer stem cells--the current status of an old concept: literature review and clinical approaches. Biol. Res..

[10] Gao C, Kong NR, Chai L (2011). The role of stem cell factor SALL4 in leukemogenesis. Crit. Rev. Oncog..

[11] Gazdar AF (2009). Activating and resistance mutations of EGFR in non-small-cell lung cancer: role in clinical response to EGFR tyrosine kinase inhibitors. Oncogene.

[12] Ghosh G, Lian X, Kron SJ, Palecek SP (2012). Properties of resistant cells generated from lung cancer cell lines treated with EGFR inhibitors. BMC Cancer.

[13] Gong Y, Pao W (2012). EGFR mutant lung cancer. Curr. Top. Microbiol. Immunol..

[14] Hanna JM, Onaitis MW (2013). Cell of origin of lung cancer. J. Carcinog..

[15] Itou J, Matsumoto Y, Yoshikawa K, Toi M (2013). Sal-like 4 (SALL4) suppresses CDH1 expression and maintains cell dispersion in basal-like breast cancer. FEBS Lett..

[16] Jeong HW (2011). SALL4, a stem cell factor, affects the side population by regulation of the ATP-binding cassette drug transport genes. PLoS One.

[17] Jia X, Qian R, Zhang B, Zhao S (2016). The expression of SALL4 is significantly associated with EGFR, but not KRAS or EML4-ALK mutations in lung cancer. J. Thorac. Dis..

[18] Kaur S, Singh G, Kaur K (2014). Cancer stem cells: an insight and future perspective. J. Cancer Res. Ther..

[19] Kobayashi D, Kuribayashi K, Tanaka M, Watanabe N (2011). Overexpression of SALL4 in lung cancer and its importance in cell proliferation. Oncol. Rep..

[20] Leung EL (2010). Non-small cell lung cancer cells expressing CD44 are enriched for stem cell-like properties. PLoS One.

[21] Liu K (2013). The multiple roles for Sox2 in stem cell maintenance and tumorigenesis. Cell Signal..

[22] Metro G, Crino L (2012). Advances on EGFR mutation for lung cancer. Transl. Lung Cancer Res..

[23] Okayama H (2012). Identification of genes upregulated in ALK-positive and EGFR/KRAS/ALK-negative lung adenocarcinomas. Cancer Res..

[24] Rad SM (2015). Transcription factor decoy against stem cells master regulators, Nanog and Oct-4: a possible approach for differentiation therapy. Tumour Biol..

[25] Siegel R, Ma J, Zou Z, Jemal A (2014). Cancer statistics, 2014. Ca. Cancer J. Clin..

[26] Stella GM (2012). Targeting EGFR in non-small-cell lung cancer: lessons, experiences, strategies. Respir. Med..

[27] Thomson S, Petti F, Sujka-Kwok I, Epstein D, Haley JD (2008). Kinase switching in mesenchymal-like non-small cell lung cancer lines contributes to EGFR inhibitor resistance through pathway redundancy. Clin. Exp. Metastas..

[28] Tricker EM (2015). Combined EGFR/MEK inhibition prevents the emergence of resistance in EGFR-mutant lung cancer. Cancer Discov..

[29] Xiong J (2014). SALL4: engine of cell stemness. Curr. Gene. Ther..

[30] Yakaboski E, Jares A, Ma Y (2014). Stem cell gene SALL4 in aggressive hepatocellular carcinoma: a cancer stem cell-specific target?. Hepatology.

[31] Yanagihara N (2015). Significance of SALL4 as a drugresistant factor in lung cancer. Int. J. Oncol..

[32] Yao Z (2010). TGF-beta IL-6 axis mediates selective and adaptive mechanisms of resistance to molecular targeted therapy in lung cancer. Proc. Natl Acad. Sci. USA.

[33] Yong KJ (2016). Targeting SALL4 by entinostat in lung cancer. Oncotarget.

[34] Yuan X (2016). SALL4 promotes gastric cancer progression through activating CD44 expression. Oncogenesis.

[35] Yue X (2015). High cytoplasmic expression of SALL4 predicts a malignant phenotype and poor prognosis of breast invasive ductal carcinoma. Neoplasma.

[36] Zeng SS (2014). The transcription factor SALL4 regulates stemness of EpCAM-positive hepatocellular carcinoma. J. Hepatol..

[37] Zhang L (2014). SALL4, a novel marker for human gastric carcinogenesis and metastasis. Oncogene.

[38] Zhang X, Yuan X, Zhu W, Qian H, Xu W (2015). SALL4: an emerging cancer biomarker and target. Cancer Lett..

[39] Zwitter M (2014). Intercalated chemotherapy and erlotinib for advanced NSCLC: high proportion of complete remissions and prolonged progression-free survival among patients with EGFR activating mutations. Radiol. Oncol..

