# Supplementary material for: Upregulation of SALL4 by EGFR activation regulates the stemness of CD44-positive lung cancer
Source: Oncogenesis. 2018 Apr 25;7(4):36. doi: 10.1038/s41389-018-0045-7 (PMC5915399; doi:10.1038/s41389-018-0045-7)
Supplement: Supplementary file 1 — Supplementary Figure legend [file 41389_2018_45_MOESM1_ESM.docx]

Supplementary S1. Knockdown of SALL4 in Bease-2B cells infected with Lenti-control or SALL4 shRNA was evaluated by western blotting.
